# How welcome do Iranian-Americans feel in their homeland? Perceptions of social distance among Muslim, Jewish, and Non-Religious Iranian-American adults

**DOI:** 10.1186/s40064-015-1531-4

**Published:** 2015-12-01

**Authors:** Shari Paige, Elaine Hatfield, Lu Liang

**Affiliations:** University of Hawaii at Manoa, 4 Midlothian, Dove Canyon, CA 92679 USA

**Keywords:** Iranian-American, Muslims, Jews, Prejudice, Social distance

## Abstract

Recent political events in the United States have created a political climate that promotes prejudice against Middle Eastern, Iranian, and Muslim people. In this study, we were interested in investigating two questions: (1) How welcome do Iranian-American men and women from various religious backgrounds (Muslim, Jewish, or no religious affiliation) feel in their new homeland (specifically, how much social distance (affective distance) do they think their Euro-American neighbors feel toward them? and (2) to what extent does the possession of stereotypical Middle Eastern, Iranian, or Muslim traits (an accent, darker skin, wearing of religious symbols, traditional garb, etc.) spark prejudice and thus Iranian-Americans perception of social distance? Participants were recruited from two very different sources: (1) shoppers at grocery stores in Iranian-American neighborhoods in Los Angeles, and (2) a survey posted on http://Surveymonkey.com. A total of 374 Iranian-Americans, ages 18 and older, completed an in-person or online questionnaire that included the following: a request for demographic information, religious preferences, a survey of how typically Iranian-American the respondents’ traits were, and the social distance scale. A surprise was that it was the Iranian-American *Jews* (not the Muslims), who felt most keenly that Euro-Americans kept them at a distance. Jewish women received higher scores on the social distance scale than did members of any other group. In addition, again, it was mainly Iranian-American Jews, particularly those who spoke with a Middle Eastern accent or wore stereotypically religious symbols, who felt the most social distance existing between them and “typical” Americans.

## Background

At one time, the USA and Iran had fairly cordial relations. When Mohammad Reza Shah Pahlavi was the Shah from 1941 until his overthrow (in 1979), the USA was a strong supporter of Iran and the Shah. In 1979, the Iranian Revolution occurred, and the Ayatollah Khomeini seized power. He was named Supreme Leader, the country was renamed the Islamic Republic of Iran, and the Iranian legal system was replaced by a set of Islamic laws and regulations. American-Iranian relations began to deteriorate. In 1979, the Iran Hostage Crisis occurred; 63 American diplomats and citizens were held prisoner in Tehran for 444 days. Starting in 1982, the USA began secretly assisting Saddam Hussein in the Iranian/Iraq war, supplying Iraq with billions of dollars in economic aid, military intelligence, and weapons. In 1988, Iranian Air Flight 655, an Iranian civilian passenger plane making a routine run from Tehran to Dubai, was shot down by the US missile Cruiser USS Vincennes in the Persian Gulf. On November 11, 2001, when al Qaeda supporters flew planes into the World Trade Center and the Pentagon in Washington, D. C., Iranian-American relations plummeted. In 2002, President George W. Bush designated Iran, Iraq, and North Korea as the “Axis of Evil” and named Iran as a terrorist state. In recent months (in 2015), President Obama’s attempts to negotiate an anti-nuclear agreement with Iran has reignited anti-Iranian passions.

“Anti-Iranian sentiment” refers to feelings and expression of hostility, hatred, prejudice directed toward Iran and its culture and toward men and women of Iranian descent. In this paper, we will define prejudice as “thinking ill of others without sufficient warrant” (Allport 1954, p. 6). “Discrimination comes about only when we deny to individuals or groups of people equality of treatment which they may wish” (Allport 1954, p. 51).

Today, condemnations of Iran and Iranian-Americans are fairly common. Conservative commentator Ann Coulter referred to Iranians as “ragheads” (Gossett 2008). Brent Scowcroft, a one-time National Security Agency advisor, called the Iranian people “rug merchants” (New York Times 2007). The *Columbus Dispatch* recently ran a cartoon portraying Iran as a sewer with cockroaches crawling out of it (NIAC 2007). Debra Cagan, a senior official at The Pentagon, declared: “I hate all Iranians” (Wikipedia 2015a, b). In March, 2015, Bolton (20l5), one time US ambassador to the United Nations, in a *New York Times* op-ed piece, advised, “To Stop Iran’s Bomb, Bomb Iran” (For a comprehensive review of the history of American-Iranian relations and anti-Iranian-American prejudice and discrimination, see Harris 2012; Paige 2014).

When two nations are at odds, resentments tend to spill over into the dialog back home. American citizens begin to resent those whose ethnic background is different from their own. The process by which people begin to hate others has been described as the “mirror image” effect (White 1970). Namely, when two countries, such as Iran and the USA, have contentious relations, the American majority will tend to label Iran and its Iranian-American citizens as bad and wrong and to justify their own country and people as good and right (Peteraf and Shanley 1997; White 1970). In the wake of the 1979 Iran Hostage Crisis, for example, 70 % of American participants reported a positive image of Americans (considering them as friendly and safe), and a negative image of Iranians (e.g., unfriendly and dangerous; Johnston et al. 1980).

People also typically report less favorable attitudes toward members of countries that are culturally dissimilar than those that are similar to themselves (Nincic and Russett 1979; Rouhana and Fiske 1995). Important cultural dimensions upon which Iran and Iranian-Americans differ from the USA are political characteristics, religion, and language. The increased likelihood of favorable attitudes toward those who are culturally similar is due to categorization of individuals as belonging to the in-group (or possessing a closer relation to the in-group) versus the out-group. The in-group is comprised of the cultural, ethnic, and social groups one feels a part of; the out-group is comprised of social groups one competes with and does not associate with (Tajfel and Turner 1979).

Categorizing people as part of the in-group versus out-group has significant psychological implications. Differences between the in-group and out-group are described as the social–cognitive perception of differences between “we and they” (McLauchlin and Pearlman 2011; Tajfel and Turner 1979; Yamagishi et al. 2008). People sympathize and feel similar to members of their in-group and dislike and compete with members of the out-group (Tajfel and Turner 1979; Waldzus et al. 2004). The out-group categorization of individuals leads to “a negative evaluation of a social group, or a negative evaluation of a person that is significantly based on the individual’s group memberships” (Crandall and Eshleman 2003, p. 414), in order to maintain a degree of cognitive consistency (Bronfenbrenner 1961; Nail et al. 2003).

The news for Iranian-Americans, however, is not all bad. Many people, such as President Obama, have praised Iranian-Americans for their patriotism, success in the United States, and hard work. Advocates point out that the United States has been welcoming to Iranian immigrants, and that prominent Iranian-Americans can be found among the ranks of diplomats, scientists, educators (Robert Mehrabian, President of Carnegie Mellon), business (Arash Ferdowsi, CEO of Dropbox; Alex Mehr and Shajan Zedeh, founders of Zoosk), filmmakers (Bob Yari, Academy Award winning film producer), NBA players (Hamed Haddadi, center for Phoenix Sons), actors, comedians, and authors (e.g., Dumas, author of the 2003 book *Funny in Farsi: A Memoir of Growing up Iranian in America,* which sold over 100,000 copies).

### Perceptions of Iranian-American men and women

Iranian-Americans are a diverse group. Although many identify with the Shi’a, Sunni, and Sufi branches of Islam, others consider themselves to be Bahá’í, Christians, Jews, Mandeans, Yarsanis, or Zoroastrians. Some are atheists. Iranian-Americans also vary greatly in education, occupation, and income. In fact, although Iranian-Americans’ yearly income, education, and occupation exceed the US national average, income varies too. In spite of that diversity, many Americans stereotype Iranian-Americans. Eagly and Kite (1987), for example, surveyed 303 students at Purdue University. The undergraduate students were asked to rate individuals from 28 countries. Participants stereotyped Middle-Eastern and Iranian-American men and women as religious, traditional, and poor. Iranian-American men were stereotyped as hostile, aggressive, never giving up, dirty, proud, and arrogant; Iranian-American women were stereotyped as family oriented, conforming, conservative, proud, devoted to others, honest, and emotional. More recently, Ghavami and Peplau (2013) surveyed 627 undergraduates from a Southern California University. They found that Middle Eastern men and women were stereotyped as Muslim, dark-skinned, and religious. Middle Eastern men were stereotyped as anti-west, suspicious, and good at bargaining. Middle Eastern women were stereotyped as quiet, covered, oppressed, family-oriented, having many children, sexually conservative, and (being) housewives.

Consequently, as a result of experiencing public disapproval, Middle Easterners and Muslims in the USA are at risk of suffering alienation, isolation, depression, and anxiety (Britto 2008; Clay 2011), and post-traumatic stress disorder (Clay 2011). Middle Easterners experience hate crimes (Human Rights Watch 2002), racial profiling (Siggins 2002), negativity during job interviews (King and Ahmad 2010), prejudice and discrimination within academia (Omeish 1999), and confront personal identity issues (Bradford 2009; Zaman 2010).

Such prejudiced attitudes sometimes spill over into hate crimes directed against Middle Easterners and anyone who is assumed to be Middle Eastern or Muslim (Britto 2008). According to the Federal Bureau of Investigation reports (Human Rights Watch 2002), anti-Middle Eastern hate crimes increased 17-fold from the year 2000 to 2001 (as cited in Human Rights Watch 2002).

### Differences among Iranian-Americans in perceptions of how accepting and rejecting the “Typical” American is towards them

Most past research has focused on either ethnicity *or* religion. However, it is the intersection of ethnicity (Middle Eastern/Iranian) and religion (Muslim), that sparks the biggest spike in prejudice and discrimination (Human Rights Watch 2002).

Not surprisingly, as prototype theory would have it (Allport 1954; Inman and Baronn 1996; Schneider 2005), people who are most easily identified as members of stigmatized groups are generally most vulnerable to victimization. Members of the Middle Eastern community who hold occupations that are more typical for Middle Easterners are often especially likely to be victims of hate crimes (Human Rights Watch 2002). For example, taxi drivers, convenience store owners, and motel owners were more likely to experience hate crimes than were those Middle Easterners who do not occupy typical occupations for their ethnic group.

Other signs of group membership (and the ones in which we are interested here) are a Middle Eastern accent, skin color, the wearing of obvious religious symbols, and dress. Dress includes a hijab for women and a turban for men in the Middle Eastern or Muslim communities. The hijab is a traditional Muslim article of clothing worn by women in the community to cover their hair. The turban is a traditional Middle Eastern (also African and Far Eastern) headwear worn by men of the community, who may belong to a number of religious groups.

### How do “Typical” Americans’ attitudes impact Iranian-American men and women?

Thus far we have focused on the attitudes of “typical” American. Our next question (and the focus of this study) is “How do typical Americans’ prejudice and discrimination against Iranian-American Muslims affect that target group?” One scale often used to describe how willing people are to engage in social contacts with other social groups (such as racial and ethnic groups), is the Bogardus (1959) social distance scale. Bogardus asks the extent to which people in various groups would be accepting of other groups (a score of 1.00 = the groups feel no social distance from one another). Groups ask to what extent they approve of the following types of intimacy with a given group. Would they admit them to:

- As close relatives by marriage (i.e., as the legal spouse of a close relative).
- As my close personal friends.
- As neighbors on the same street.
- As co-workers in the same occupation.
- As citizens in my country.
- As non-citizen visitors in my country.
- Would exclude from entry into my country.

The Bogardus social distance scale is a cumulative scale (a Guttman scale), because agreement with an earlier item (say, would accept in marriage) implies agreement with the following items (i.e., would, say, admit as citizens to the country).

On the basis of the above theorizing, we proposed a series of hypotheses and questions:

### Hypotheses

We assume that all Middle-Easterners, be they religious or non-religious, male or female, easily identifiable or noticeable only to the few, might expect to encounter some prejudice. In our study, we planned to explore the factors that make men and women especially susceptible to feeling welcome/unwelcome in their adopted homeland. Specifically:

#### **Hypothesis I**

Gender and religious affiliation (Muslim, Jewish, or no religious affiliation) will affect Iranian-Americans’ perceptions as to how willing Euro-Americans are to welcome them. Specifically, Iranian-American *Muslims* will secure higher scores on the Social Distance Scale than will their non-Muslim peers (be they Jewish or those possessing no religious affiliation). Muslim men will score higher on the Social Distance Scale than will Muslim women.

#### **Hypothesis II**

Iranian-American *Muslims* who possess a strong Iranian accent, darker skin color, or who appear prototypically Muslim, engage in displays of religious affiliation or wear ethnically traditional clothing will secure higher scores on the Social Distance Scale than will Iranian-American Muslims whose appearance is more prototypically Euro-American. We will also consider generational status, identification with an ethnicity in addition to Iranian-American, family income, and education (as other possible markers of assimilation). We plan to test Hypothesis II by exploring two separate questions:

#### **Question 1**

Are Iranian-Americans’ perceptions of Euro-Americans’ social distance *within the full sample* predicted by Muslim identity, Jewish identity, gender, Iranian accent, skin color, displays of religious affiliation, displays of ethnically traditional clothing, generational status, identification with an ethnicity in addition to Iranian-American, family income, and education?

#### **Question 2**

Are Iranian-Americans’ perceptions of Euro-Americans’ social distance *within each religious subsample* (Iranian-Americans: Muslim, Jewish, no religious affiliation), predicted by gender, Iranian accent, skin color, displays of religious affiliation, displays of ethnically traditional clothing, generational status, identification with an ethnicity in addition to Iranian-American, family income, and education?

To answer these questions we conducted the following study.

## Method

### Participants

This study was described to potential participants as follows: “A Ph.D. student in the Department of Psychology at the University of Hawai’i, Mānoa is conducting a study on the social attitudes of Iranian-Americans. We are interested in perceptions of Iranian-Americans as to the extent to which prejudice and/or discrimination against Iranian-Americans exists in the USA You are being asked to participate because you are an adult over the age of 18 who identifies as Iranian-American”.

Participants consisted of 341 Iranian-Americans [111 men (33 %) and 230 (67 %) women], ranging in age from 18 to 68 [*M* = 38, *SD* = 11.67]. A full 44 % identified as Muslim, 24 % identified as Jewish, 31 % reported having no religious affiliation, and the rest were Christian, other, or did not report their religion. Education varied as follows: grade school (2 %), high school (12 %), vocational degree or certification (4 %), BA/BS (34 %), MS/MA (24 %), or Ph.D./MD (15 %). A few failed to indicate their educational level (9 %). When asked what their annual household income was during childhood, participants indicted the following: Under $17,000 (4 %), $17,000–$24,999 (5 %), $25,000–$49,999 (13 %), $50,000–$99,999 (37 %), and $100,000 + (29 %), with 13 % choosing not to report.

Forty-two percent of participants were interviewed face-to-face and 58 % were recruited online. The in-person participants were approached at two ethnically Persian (Iranian) grocery stores, Jordan Market and Super Sun, on Westwood Boulevard in West Los Angeles. The first author, who speaks Farsi, conducted all of these interviews.

Iranian-American participants who completed an online survey were recruited in a variety of ways. We collaborated with the Persian American Society for Health Advancement (PASHA—a non-religious organization which includes Jewish, Muslim, and Iranian-Americans with various religious affiliations). We also contacted the following Iranian-American organizations and asked them to distribute information about the survey. These were: University of Maryland Iranian Student Foundation, Persian American Association of Northern California, Iranian-American Women’s Foundation, Persian Student Association at Stanford University, Iranian Student Alliance in America at UC Berkeley, Association of Professors and Scholars of Iranian Heritage, Iranian Students Association at Arizona State University, Pars Times, Iranian-American Cultural Association of Missouri, Iranian-American Bar Association, Persian Academic and Cultural Student Association at the University of Southern California, and The Persian American Society for Health Advancement. We also sent invitations to synagogues. The following synagogues were contacted: Chabad of Bel Air, Beth Jacob Congregation, Congregation Magen David of Beverly Hills, Young Israel of North Beverly Hills, LeoBaeck Temple, and University Synagogue. The aforementioned synagogues were emailed because they were located in or around the Beverly Hills area. According to Montagne (2006) of National Public Radio, 20 % of individuals living in Beverly Hills are Iranian-American and 40 % of students who attend schools in the area are Iranian-American. Alas, most did not reply. Finally, we contacted individuals with ethnically Iranian names and invited them to complete the survey via http://LinkedIn.com. Surveys were conducted on and http://SurveyMonkey.com and were available via http://Facebook.com and http://LinkedIn.com.

### Demographics

*Ethnic background* Our first step was to ensure that all participants were Iranian-American. All were. In order to gain a fuller picture of participants’ backgrounds, we asked a few supplementary questions. These included: “Do you identify with any other ethnic group besides Iranian-American? (e.g., Azerbaijani, Afghani, Bahrani, etc.)?” About half of participants identified with an additional sub-ethnic group (say, for example, Turkish-Iranian). Participants’ generational status was also assessed. We asked, “How many generations has your family been in the USA?” The majority (83 %) was born outside the US and 18 % were born in the USA.

*Religious affiliation* Participants were asked, “What is your religious affiliation? Please write none if you have no religious affiliation.” Of the participants who were willing to disclose their religion, 44 % identified as Muslim, 24 % identified as Jewish, 31 % reported having no religious affiliation, and 1 % indicted other.

#### Independent variables

We asked a series of questions in order to ascertain how “typically” (i.e., stereotypically) Middle Eastern, Iranian-American, or Muslim participants would appear to be from their accent, skin tone, or appearance. We included the following questions:

*Accent.* Participants were asked, “Do you consider yourself to have an Iranian accent when speaking English?” Possible responses ranged from 1 = not at all to 7 = acute. A full 32 % checked “Not at all”, 13 % indicated between “Not at all” and “mild”, 15 % reported “mild”, 5 % reported an accent between “mild” and “moderate”, 17 % reported “moderate”, 12 % reported between “moderate” and “acute”, 6 % reported having an “acute” accent. The higher the score, the stronger the Iranian the accent is.

*Skin tone* Participants were asked to match their own skin tone to exemplars on The Fitzpatrick Scale, a color chart depicting various skin types (Daniel et al. 2009). Possible hues ranged from Type 1 (Red and blonde hair, blue eyes, burns easily, never tans, freckles, very fair skin) to Type 6 (Black hair, dark brown eyes. May never burn). The majority of participants (61 %) rated their skin tone as a 4 on the Fitzpatrick Scale (i.e., as possessing “dark brown hair and green, hazel, or brown eyes. Slow to burn, tans easily”). The higher the score, the darker the participant’s skin tone is.

*Symbols of religious affiliation* Participants were asked, “Do you wear symbols of your religious affiliation? (e.g., Cross, Yarmulke, Turban, Scarf, etc.).” Possible answers ranged from 0 = no to 1 = yes (i.e., sometimes or always). Most participants reported “never” displaying religious symbols (71 %), others reported “sometimes” or “always” displaying such symbols (29 %). In coding, possible answers ranged from 0 = no to 1 = yes.

Next, participants who wore such symbols were asked: “If so, what do you wear?” The majority reported wearing a scarf/hijab (15 %), displaying the Star of David (3 %), a yarmulke (2 %), a *faravahar* (symbol of Zoroastrianism) (1 %), an Allah necklace (1 %), or religious jewelry (5 %). Less than 2 % of participants reported displaying: a Kara (steel bangle worn by Sikhs) (0.3 %), evil eye jewelry (0.3 %), a cross (0.3 %), a beard (0.3 %), an Islamic Stone (0.3 %), or *tasbih* prayer beads (0.5 %).

*Ethnically traditional clothing* Participants were asked, “Do you wear ethnically traditional clothing? (e.g., clothing worn in non-urban areas of Iran, such as Mahali clothing)?” The majority (94 %) of participants reported “never” wearing such clothing. A small percentage of participants (5 %) reported “sometimes” wearing such clothing. No participant reported “always” wearing such clothing. Scores ranged from 0 = no to 1 = yes.

Those who wore such clothing were asked when they wore such clothing. Answers were assigned to the following categories: seldom (2 %), on Halloween (1 %), ethnic/cultural events (2 %), once a month (1 %), or very often (1 %). When asked what kind of ethnic clothing they wore, they indicated the following: *thobe/dishdasha* (0.3 %), *mahali* dress (1.1 %), colorful scarf (0.8 %), scarfs/jewelry (0.3 %), or bags/scarves/jewelry/skirt (1.6 %).

*Occupation* Participants were asked: “What is your occupation? We then converted specific occupations to more general categories. Answers (after our conversion) were: white collar (84 %), blue collar (1 %), homemaker (10 %), or other (5 %). In order to gain a better understanding of participants’ backgrounds, we asked two additional questions: “What was your father’s occupation?” Again, following the same procedure, specific responses were placed in the following categories: White collar (75 %), blue collar (3 %), Army (8 %), or other (14 %). “What was your mother’s occupation?” Again, participants’ responses were placed in the following categories: White collar (36 %), blue collar (0 %), homemaker (35 %), or other (29 %).

*Geographic location* Participants were asked to indicate their geographic location. Participants came from the following areas: Los Angeles, CA (28 %), Orange County, CA (28 %), California (30 %), Orange County or Los Angeles (1 %), or other (13 %).

#### Dependent variable

The Social Distance Scale (Bogardus 1959) is designed to assess Iranian-Americans’ perceptions as to the extent that Euro-Americans accept them. Our slight revision of the scale asked Iranian-Americans how accepting they think Euro-Americans are of Iranian-Americans. Possibilities range from: “Do you believe Euro-Americans would marry into the Iranian-American group?… like them as close friends?… Like them as next door neighbors?” And so forth. (see Table 1 for a description of the scale used in this study). Participants were asked to answer each question with either a yes or no. Questions 1–4 were coded as no = 1 and yes = 0. Questions 5–7 were coded as no = 0 and yes = 1. Possible (original) scores ranged from 0 to 7. Replies were averaged; averaged scores ranged from 0 to 1. The higher the number, the more convinced Iranian-Americans were that Euro-Americans would reject them—i.e., that Euro-Americans would wish to maintain a greater social distance. Cronbach’s α level for the Social Distance Scale was α = 0.82 (Angermeyer et al. 2003).

**Table 1:** Social distance scale

1. Do you believe Euro-Americans would marry into the Iranian-American group	Yes, no
2. Do you believe Euro-Americans would like Iranian-Americans as close friends	Yes, no
3. Do you believe Euro-Americans would like Iranian-Americans as next door neighbors	Yes, no
4. Do you believe Euro-Americans would like to work in the same office with Iranian-Americans	Yes, no
5. Do you believe Euro-Americans would like Iranian-Americans as speaking acquaintances only	Yes, no
6. Do you believe Euro-Americans would like Iranian-Americans as visitors only to the USA	Yes, no
7. Do you believe Euro-Americans would like to debar Iranian Americans from the USA	Yes, no

Bogardus (1959)

The higher the score, the less welcome people feel in the USA

Since its introduction in 1925, Bogardus’s scale has been subject to serious challenges, especially when used outside the Western cultural context. Weinfurt and Moghaddam (2001), for example, report that Indian and Algerian respondents deviated substantially from the assumption of ordinal scales of the questionnaire. Although results need to be interpreted with caution, scholars such as Klicperová-Baker and Kost’ál (2011) have concluded, on the basis of an extensive review of the SDS literature:

Over the years, social distance proved to be a viable concept and the SDS method is still both a favorite tool and an inspiration for constructing related social distance techniques (p. 3).

Thus we chose to use a slight variation of this traditional scale, which seemed appropriate for our Iranian-American population (see Table 1).

### Procedure

We recruited face-to-face participants from grocery stores 2 weeks prior to Persian New Year because many Iranian-Americans shop for groceries right before the holidays. Dr. Paige approached participants by introducing herself in Farsi. She then translated questions into Farsi or English, as the respondents preferred. The survey was completed with paper and pen. Participants who were recruited online were provided with a link to Surveymonkey.com.

The surveys included a consent form and the following scales: (1) a demographic questionnaire (which asked about respondents’ gender, age, ethnic background, and religion). They were also asked about the possession of a foreign accent, generational status, skin tone/color, displays of religiously symbolic clothing, displays of ethnically traditional clothing, highest level of education, family income during childhood, occupation, father’s occupation, mother’s occupation, and geographic location). The questions were randomized whenever possible to control for ordering effects.

## Results

### **Hypothesis I**

Gender and religious affiliation (Muslim, Jewish, or no religious affiliation) will affect Iranian-Americans’ perceptions as to how willing Euro-Americans are to welcome them. Specifically, Iranian-American *Muslims* will secure higher scores on the Social Distance Scale than will their non-Muslim peers (be they Jewish or those possessing no religious affiliation). Muslim men will score higher on the Social Distance Scale than will Muslim women.

Table 1 provides the descriptive statistics that allow us to explore the relationship between gender and religious affiliation and Iranian-Americans’ perceptions that Euro-Americans desire to maintain a great deal (or little) Social Distance. To our surprise, both men and women, from a variety of religions, perceived that “typical” Americans were fairly accepting of them re: Social Distance (see Table 2).

**Table 2:** Mean and standard deviation for gender and religion affiliation

Gender	Religion affiliation											
	No religion			Jewish			Muslim			Total		
	M	SD	N	M	SD	N	M	SD	N	M	SD	N
Men	0.21	0.30	52	0.26	0.26	24	0.22	0.30	34	0.22	0.29	110
Women	0.19	0.24	55	0.38	0.37	58	0.24	0.27	115	0.26	0.30	228
Total	0.20	0.27	107	0.34	0.34	82	0.23	0.28	149	0.25	0.30	338

**Fig. 1:**
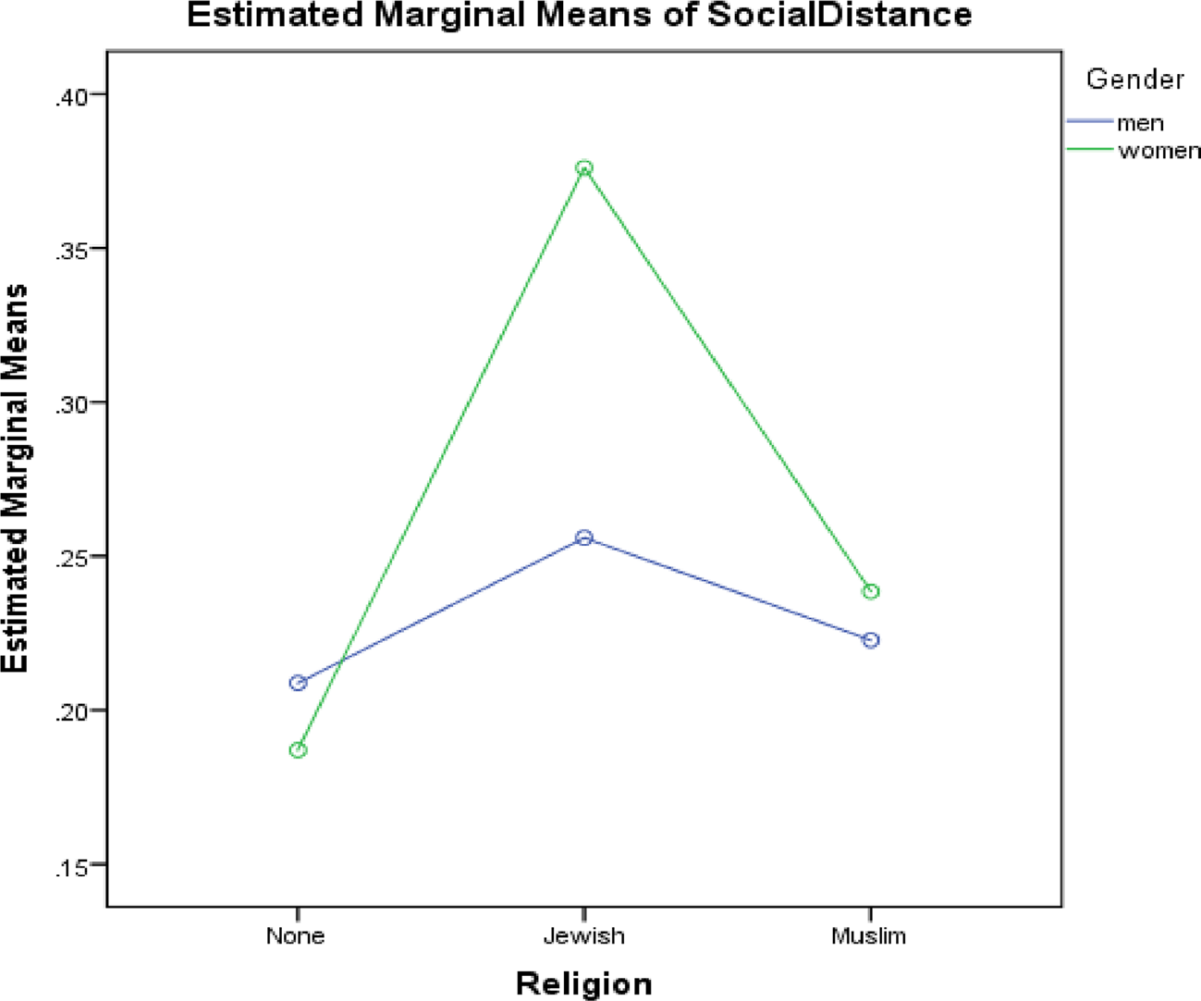
Mean social distance score by gender and religion affiliations

We conducted a two-way ANOVA to explore whether or not there were significant effects of gender and/or religion on perceptions of social distance. Results indicate that there were no statistically significant interactions between the effects of gender and religious affiliation on perceptions of social distance, *F* (2,332) = 1.27, *p* = 0.28. Figure 1 shows the mean perception of social distance score for each combination of gender group and religion affiliation group plotted in a line graph.

Main effects analysis make it clear that there was no significant effect for gender on perceptions of social distance, *F* (1, 332) = 1.15, *p* = 0.29. However, there were significant differences between the various religion affiliation groups on perceptions of Social Distance, *F* (2, 332) = 3.50, *p* = 0.03, effect size *η*^*2*^ = 0.02. A Tukey post hoc test revealed that the Jewish group (*M* = 0.34) scored significantly higher than the no religion group (*M* = 0.20, *p* = 0.00) and the Muslim group (*M* = 0.23, *p* = 0.02). There was no significant difference between the no religion and the Muslim groups on perceptions of social distance, *p* = 0.57.

Although there was no significant interaction in the two-way ANOVA, different results were secured when the effects of religious affiliation on perceptions of social distance were analyzed for gender separately. For women, there were significant differences between the various religious affiliation groups on perceptions of social distance, *F* (2, 225) = 6.63, *p* = 0.00, effect size *η*^*2*^ = 0.004. A Tukey post hoc test revealed that the Jewish women group (*M* = 0.26) scored significantly higher than the no religion women group (*M* = 0.21, *p* = 0.00) and the Muslim women group (*M* = 0.22, *p* = 0.01). There was no significant difference between the no religion women group and the Muslim women group on perceptions of social distance, *p* = 0.53. For men, no significant differences between religion affiliation groups on perceptions of social distance were found, *F* (2, 107) = 0.22, *p* = 0.80.

### **Hypothesis II**

Iranian-American *Muslims* who possess a strong Iranian accent, darker skin color, or who appear prototypically Muslim, engage in displays of religious affiliation, or wear ethnically traditional clothing will secure higher scores on the Social Distance Scale than will Iranian-American Muslims whose appearance is more prototypically Euro-American. We will also consider generational status, identification with an ethnicity in addition to Iranian-American, family income, and education (as other possible markers of assimilation).

### **Question 1**

Are Iranian-Americans’ perceptions of Euro-Americans’ social distance *within the full sample* predicted by Muslim identity, Jewish identity, gender, Iranian accent, skin color, displays of religious affiliation, displays of ethnically traditional clothing, generational status, identification with an ethnicity in addition to Iranian-American, family income, and education?

First, a bivariate correlation was used to address relationships between our predictors and our outcome variable, social distance. When the variables were continuous variables, a Pearson correlation was conducted; when the variables were categorical variables, the point-biserial correlation was conducted. Table 3 shows the correlations we secured. The results make it clear that perceptions of social distance were significantly correlated with Iranian accent, displays of religious affiliation, generational status, and sub-ethnic identification.

**Table 3:** Correlations

Variables	1	2	3	4	5	6	7	8
Skin tone	1							
Iranian accent	0.01	1						
Displays of religious affiliation	−0.10	0.31**	1					
Ethnically traditional clothing	−0.05	−0.09	0.02	1				
Generations status	0.02	0.44**	0.16**	−0.06	1			
Sub ethnic identification	0.13*	−0.41**	−0.33**	0.15**	−0.12**	1		
Family income	−0.08	0.03	−0.02	−0.03	−0.13*	−0.36**	1	
Education	0.06	−0.10	−0.19**	0.08	0.14*	0.20**	−0.14*	1
Social distance	−0.09	0.27**	0.22**	−0.08	0.18**	−0.20**	−0.10	−0.05

* *p* < 0.05; ** *p* < 0.01

Secondly, we conducted a multiple linear regression to test the predictive strength of the following predictors: skin tone (color), Iranian accent, displays of religious affiliation, ethnically traditional clothing, generational status, sub-ethnic identification (other than Iranian-American), family income, and education on average reports of perceived social distance from Euro-American. Table 4 presents these results. The eight predictors combined accounted for 13.8 % of social distance variance, *F* (8, 257) = 5.13, *p* = 0.00, and the effect size was 0.16. Iranian accent was significantly, and positively, associated with perception of social distance (*t* = 2.31, *p* = 0.02); displays of religious affiliation was significantly, and positively, associated with perception of social distance positively (*t* = 2.69, *p* = 0.01). Skin tone was negatively associated with social distance, although not significantly so, *p* = 0.07.

**Table 4:** Prediction of social distance by predictors

Model	*B*	*SE B*	*β*	*t*	*p*
Constant	0.33	0.14	–	–	–
Skin tone	−0.04	0.02	−0.11	−1.81	0.07
Iranian accent	0.02	0.01	0.16	2.31	0.02
Displays of religious	0.12	0.04	0.17	2.69	0.01
Affiliation^a^					
Ethnically traditional	−0.11	0.08	−0.09	−1.48	0.14
Clothing^b^					
Generational status^c^	0.06	0.05	0.07	1.07	0.28
Sub ethnic	−0.04	0.04	−0.07	−0.92	0.36
Identification^d^					
Family income	−0.03	0.02	−0.10	−1.52	0.13
Education	0.01	0.02	0.06	0.96	0.34
Total	*F* = 5.13, *p* = 0.00				

^a^0 = no, 1 = yes

^b^0 = no, 1 = yes

^c^0 = born in USA, 1 = born in somewhere else

^d^0 = no, 1 = yes

### **Question 2**

Are Iranian-Americans’ perceptions of Euro-Americans’ social distance *within each religious subsample* (Iranian-Americans: Muslim, Jewish, no religious affiliation), predicted by gender, Iranian accent, skin color, displays of religious affiliation, displays of ethnically traditional clothing, generational status, identification with an ethnicity in addition to Iranian-American, family income, and education?

A multiple linear regression was conducted for Jewish (Table 5), Muslim (Table 6), and no religion group (Table 7) separately to compare religious affiliation differences on predicting perceptions of social distance by the eight predictors. For Jewish group, Iranian accent and displays of religious affiliation predicted perceptions of social distance positively, *t* = 3.47, *p* = 0.00, and *t* = 2.94, *p* = 0.01, separately. For Muslim group, none of the predictors predicted perceptions of social distance significantly. For no religion group, skin tone predicted perceptions of social distance negatively, *t* = −2.50, *p* = 0.01. For the no religion group, generational status is positively associated with social distance, although not significantly so, *p* = 0.07.

**Table 5:** Prediction of social distance by predictors for Jewish Group

Model	*B*	*SE B*	*β*	*t*	*p*
Constant	0.21	0.37	–	–	–
Skin tone	−0.01	0.06	−0.01	−0.09	0.93
Iranian accent	0.07	0.02	0.44	3.47	0.00
Displays of religious	0.26	0.09	0.34	2.94	0.01
Affiliation^a^					
Ethnically traditional	−0.09	0.21	−0.05	−0.46	0.65
Clothing^b^					
Generational status^c^	0.02	0.12	0.02	0.16	0.87
Sub ethnic	−0.01	0.16	−0.01	−0.08	0.94
Identification^d^					
Family income	−0.05	0.05	−0.11	−0.92	0.36
Education	−0.01	0.04	−0.02	−0.16	0.87
Total	*F* = 5.36, *p* = 0.00				

^a^0 = no, 1 = yes

^b^0 = no, 1 = yes

^c^0 = born in USA, 1 = born in somewhere else

^d^0 = no, 1 = yes

**Table 6:** Prediction of social distance by predictors for Muslim Group

Model	*B*	*SE B*	*β*	*t*	*p*
Constant	0.43	0.27	–	–	–
Skin tone	−0.03	0.03	−0.08	−0.74	0.46
Iranian accent	0.00	0.02	0.01	0.11	0.91
Displays of religious	0.06	0.07	0.10	0.82	0.41
Affiliation^a^					
Ethnically traditional	−0.23	0.16	−0.15	−1.45	0.15
Clothing^b^					
Generational status^c^	0.01	0.09	0.01	0.10	0.92
Sub ethnic	−0.05	0.07	−0.09	−0.76	0.45
Identification^d^					
Family income	−0.01	0.03	−0.03	−0.24	0.81
Education	−0.01	0.02	−0.06	−0.53	0.60
Total	*F* = 1.08, *p* = 0.39				

^a^0 = no, 1 = yes

^b^0 = no, 1 = yes

^c^0 = born in USA, 1 = born in somewhere else

^d^0 = no, 1 = yes

**Table 7:** Prediction of social distance by predictors for no religion group

Model	*B*	*SE B*	*β*	*t*	*p*
Constant	0.52	0.21	–	–	–
Skin tone	−0.08	0.03	−0.27	−2.50	0.01
Iranian accent	−0.01	0.02	−0.04	−0.37	0.72
Displays of religious	0.20	0.11	0.19	1.80	0.08
Affiliation^a^					
Ethnically traditional	−0.07	0.09	−0.08	−0.80	0.43
Clothing^b^					
Generational status^c^	0.14	0.08	0.21	1.87	0.07
Sub ethnic	−0.04	0.08	−0.05	−0.46	0.65
Identification^d^					
Family income	−0.02	0.03	−0.10	−0.91	0.37
Education	0.00	0.03	0.02	0.15	0.88
Total	*F* = 2.17, *p* = 0.04				

^a^0 = no, 1 = yes

^b^0 = no, 1 = yes

^c^0 = born in USA, 1 = born in somewhere else

^d^0 = no, 1 = yes

## Discussion

### Summary of key findings

There were several surprises in our data. We discovered far more than we had bargained for. In Hypothesis I we proposed that it would be the Iranian-American *Muslims* who would perceive the most prejudice—i.e., that Euro-Americans were most eager to keep them at a distance.^1^ This hypothesis was not supported. In Hypothesis II we further proposed that it would be the Iranian-American *Muslims,* who appeared prototypically Muslim (e.g., displaying scarf/hijab, Allah necklace, a beard, Islamic stone, or a Tasbih), who would perceive that Euro-Americans wanted to keep them at a distance. Again we were wrong.

In contrast to Hypothesis I, it was Iranian-American *Jews* who perceived that Euro-Americans desired to keep them at a distance. How can we account for this unexpected finding? We can think of several possible explanations.

Perhaps Iranian-American Muslims are more socially insulated than are Iranian-American Jews… and thus are unaware of the “typical” American’s prejudices. Theorists have argued that individuals who are members of historically stigmatized groups (like the Jews in America) are vigilant for signs of prejudice and often have a “trigger finger” when it comes to recognizing prejudice (Allport, 1954).

Perhaps Jews feel particularly vulnerable in the current American climate—where Netanyahu and the State of Israel and many Republican conservatives are at loggerheads with Obama and the Iran treaty.

Another explanation (for the fact that Jewish women are especially likely to perceive social distance between them and the Euro-American community) may have to do with Jewish women’s traditional roles as wives and mothers. One of the many components of traditional “women’s work” has been to maintain the emotional and personal well being of one’s family (Hochschild and Machung 2012). Perhaps (as a minority within a minority) Iranian-American Jewish women feel it is their responsibility to transmit their Jewish culture inter-generationally as a way of maintaining the community’s health, well-being, and overall existence. Thus, they may feel compelled to marry and maintain strong relationships with other Iranian-American Jews—resulting in a higher degree of perceived social distance from Euro-Americans.

Another surprise: Americans’ preference for lighter skin tones and the advantages it confers on the possessors have been widely documented in the past (Jones, 2000). Interestingly, however, the analysis revealed that although skin color mattered for Iranian-American Jews and Muslims, *the correlation between skin color and social distance was in the opposite direction from that we had proposed.* Iranian-American Jews and Muslims with lighter skin tones perceived that Euro-Americans desired greater social distance than did their darker skinned peers. How can this be? There are several possibilities.

Perhaps Iranian-Americans with lighter skin tones are assumed to be Euro-American. Thus, Euro-Americans my freely express their prejudices toward Middle Easterners in the presence of light skinned Iranian-Americans. Discovering how typical Americans *really* feel about Middle-Easterners may make Iranian-Americans aware of the gulf that exists between themselves and the typical American. A second possibility is that lighter skinned Iranian-American Muslims and/or Jews may feel rejected by both the mainstream Euro-Americans and by their own (darker skinned) peers. In-group members may assume that they do not experience much prejudice or discrimination and thus resent them for their alleged privilege.

Past researchers have demonstrated higher rates of prejudice and discrimination toward individuals with foreign accents (Lippi-Green 1994). Again, contrary to predictions, Iranian-American Jews perceived the greatest social distance from Euro-Americans, based on acuteness of foreign accent—more so than Iranian-American Muslims or Iranian-Americans with no religious affiliation. Again, this may be because Iranian-American Jews contend with layers of prejudice and discrimination based on their ethnicity, religion, and accent. Also, there are numerically fewer Iranian-American Jews in America and the world (than Iranian-American Muslims or those without religious affiliation). This may amplify Iranian-American Jewish perceptions of social distance.

In the current study we created categories for Islamic and Jewish appearance. Iranian-American Jews were the only group that reported significantly higher rates of perceived prejudice when displaying symbols of religious affiliation. In addition, both Iranian-American Jewish men and women with Jewish appearances (displaying the Star of David or a Yarmulke) reported significantly higher rates of perceived prejudice from Euro-Americans than Iranian-American Muslims and those with no religious affiliation.

Lastly, we found that Iranian-Americans who were born outside of the USA perceived higher rates of social distance than did those who were born in the USA One may suspect that Iranian-Americans born in the USA may be more assimilated into USA society than are their peers. It may be the case that cultural assimilation shields some Iranian-Americans from experiencing higher rates of perceived social distance from Euro-Americans.

### Strengths and limitations

Dr. Paige speaks both English and Farsi with native fluency. This aided with translation and avoiding cultural mistrust issues. It also helped us secure endorsements and collaborative efforts from Iranian-American organizations.

There are some limitations on our study, of course. Ideally, we would have interviewed a random sample of Iranian-Americans living in the United States. This was impossible for economic reasons, of course. While conducting the study in Southern California was the next best thing, since it afforded access to a large number of Iranian-Americans. Large populations of Iranian-Americans reside in the areas of Orange County (the city of Irvine) and Los Angeles. In fact Los Angeles has the largest number of Iranians outside of Iran. The majority of Iranian-American Jews in the study were recruited from West Los Angeles. The majority of Iranian-American Muslims were recruited from Orange County. It is unclear if there are more or less Iranian-American Muslims in Orange County verses Los Angeles, since the past researchers have only inquired about respondents’ ethnicity.

Recruiting from this region came with some drawbacks, however. First off, both of these Southern California regions are affluent and median incomes far exceed national averages [OC, *Mdn* = 75,762, Century City (West LA), *Mdn* = 95,135, National average = 42,979.61].

Iranian-Americans living in the Southern California region reside in a state that predominantly votes for the Democratic Party in elections. *The LA Times* described Southern California as a “melting pot”, with diverse communities and neighborhoods that represent a number of racial ethnic groups. Despite the general political and cultural characteristics of California, Orange County is heavily populated with a majority of Republicans. Nevertheless, the state is heavily populated with immigrants and is inclusive of cultures from many parts of the world. It is unclear how the political and economic makeup of this region affects the experiences of Iranian-Americans.

Another challenge was that although most Iranian-Americans surveyed were born outside the USA, we failed to ask if they were refugees. The psyche, mentality, and culture of a refugee are significantly different from that of an immigrant.

Some of the unique challenges we faced in surveying Iranian-Americans were cultural mistrust, refusal to denote religious affiliation, and the fact that most participants were immigrants or refugees. While conducting the current study we found that many Iranian-Americans were extremely suspicious of how the information would be utilized. Many asked if there would be governmental tracking. Upon further conversation many participants voiced a concern. They described how in Iran, government officials would ask about political attitudes in a seemingly safe environment, only to persecute individuals who had opinions against the regime. As a result, many participants worried that a similar strategy could be employed in the USA Thus, many individuals declined to take the survey and many others were very hesitant about answering questions about social distance from Euro-Americans. We assured participants, who did complete the survey that it was confidential and that the only general demographic information that could be derived from our published papers.

Another interesting finding was that many Iranian-Americans did not want to disclose their religious affiliation. Perhaps this refusal was due to fear of social marginalization or discrimination. This was evident by a few participants who reported not having any religious affiliation but “sometimes” (6 %) or “always” (2 %) displaying symbols of religious affiliation. Perhaps these individuals perceived religion as part of their ethnic identity and did not make a religious distinction within their personal identity. Hence some participants verbally identified as Iranian-American Jewish without being asked about religious affiliation. This area requires additional research.

Another challenge was that most Iranian-Americans surveyed were born outside the USA and we failed to ask if they were refugees. The psyche, mentality, and culture of refugees is significantly different from that of an immigrant.

Unfortunately, the UH IRB required us to reveal (on the consent form) our interest in studying the social attitudes of Iranian-Americans, specifically their perceptions of the extent to which prejudice and/or discrimination against Iranian-Americans exists in the USA. Thus, by directly revealing what we aimed to measure, we may have attracted individuals who (1) perceived that “typical” Americans kept them at a distance, and/or (2) who were emotionally ready to talk about their perceptions. And, of course, since the 1960 s, social psychologists such as and Carl Hovland, Irving Janis, and Daniel Katz have documented that if people’s group membership is made salient (even implicitly), they are far more likely to express stereotyped attitudes than when simply asked about their own opinions (Fiske et al. 1987).

Participants were given a questionnaire written in English. If respondents were more comfortable speaking Farsi, the first author translated the items for them. Naturally, researchers always worry that participants’ ethnicity and native language might affect their responses (Ervin-Tripp 2000; Nesbitt 2004). Whether or not groups differed in this way is, of course, a question for another study.

Researchers always face challenges in determining wither participants’ perceptions match observable facts.

### Suggestions for subsequent research

It would be interesting to access the degree of participants’ assimilation into the USA culture and society. We need to determine whether those who fit in are more aware of others’ prejudices or feel less prejudice directed toward themselves.

We should also “unpack” what is included in the simple religious labels “Muslim”, “Jewish”, or “No religion.” Obviously, people in those categories may differ in a wide variety of other ways. When did the families come to America? How insular are the various groups at the present time (for example, do the “insular” Muslim groups have greater intermarriage rates or live in enclaves?) How affluent are the different groups? What is their social class? What is their political affiliation? How sympathetic or opposed are they to the current Iranian political groups? Do they support the current Peace treaty? Which religious affiliations are seen as most “American?” From the data, we might expect Americans, with Judeo Christian roots, see the “no religion” participants as most similar to themselves—since this group reports less awareness of prejudice (social distance).

It would be interesting for future researchers to explore what (if any) social, political, or economic benefits or privileges Iranian-Americans gain through assimilation, taking into account religious affiliation.

Must Iranian-American Muslims and Jews change their religious affiliation in order to assimilate? These are all questions we hope to explore in future studies.

## Concluding comments

The current study demonstrated the complex social identities of Iranian-Americans. As is evident by the results enumerated above, Iranian-American perceptions (and perhaps actual experiences) are significantly influenced by religious affiliation. We hope that researchers will begin to examine Iranian-American identity in a more multi-dimensional manner that takes into account the intersectionality of ethnicity and religion. The study also demonstrated that Iranian-American Jews and Muslims are vulnerable and perceive higher rates of social distance from Euro-Americans. Thus, this is an issue that should be taken into account when developing policy and standards of practice both in public and private organizations.

## Footnotes

1 We are using the term “prejudice” here to indicate the perception that Euro-Americans prefer to keep their distance from Iranian-Americans. The terms have a somewhat different meaning, of course. We are making the substitution because to use the long phrase (above) gets annoying. We assume readers will know what we mean.

## References

[CR1] Abrams D, Wetherell M, Cochrane S, Hogg MA, Turner JC (1990). Knowing what to think by knowing who you are: self-categorization and the nature of norm formation, conformity, and group polarization. Br J Soc Psychol.

[CR2] Allport GW (1954). The nature of prejudice.

[CR3] Angermeyer MC, Matschinger H, Corrigan PW (2003). Familiarity with mental illness and social distance from people with schizophrenia and major depression: testing a model using data from a representative population survey. Schizophr Res.

[CR4] Bogardus ES (1959). Social distance.

[CR5] Bogardus ES (1967). A forty year racial distance study.

[CR6] Bolton JR (20l5) The opinion pages: to stop Iran’s bomb, bomb Iran. New York Times. http://www.nytimes.com/2015/03/26/opinion/to-stop-irans-bomb-bomb-iran.html

[CR7] Bradford JW (2009). American/Muslims: reactive solidarity, identity politics and social identity formation in the aftermath of September 11th. Diss Abstr Int Sect.

[CR8] Britto PR (2008). Who am I? Ethnic identity formation of Arab Muslim children in contemporary US society. Am Acad Child Adolesc Psychiatry.

[CR9] Bronfenbrenner U (1961). The mirror-image in soviet american relations: a social psychologist’s report. J Soc Issues.

[CR10] Clay RA (2011). Muslims in America, post 9/11. Am Psychol Assoc.

[CR11] Crandall CS, Eshleman A (2003). A justification-suppression model of the expression and experience of prejudice. Psychol Bull.

[CR12] Daniel LC, Heckman CJ, Kloss JD, Manne SL (2009). Comparing alternative methods of measuring skin color and damage. Cancer Causes Control.

[CR13] Dumas F (2003). Funny in Farsi: A memoir of growing up Iranian in America.

[CR14] Eagly AH, Kite ME (1987). Are stereotypes of nationalities applied to both women and men?. J Pers Soc Psychol.

[CR15] Ervin-Tripp S (2000). Bilingual minds. Bilingualism.

[CR016] Fiske ST, Neuberg SL, Beattie AB, Milberg SJ (1987). Category-based and attribute-based reactions to others: Some informational conditions of stereotyping and individuating processes. J Exp Soc Psychol.

[CR16] Ghavami N, Peplau LA (2013). An intersectional analysis of gender and ethnic stereotypes. Testing three hypotheses. Psychol Women Q.

[CR17] Gossett S (2008) Ann Coulter ‘Raghead’ comments spark blogger backlash. CBS News.com. http://www.cnsnews.com/news/article/ann-coulter-raghead-comments-spark-blogger-backlash

[CR18] Gryczynski J, Ward BW (2012). Religiosity, heavy alcohol use, and vicarious learning networks among adolescents in the United States. Health Edu Behav.

[CR19] Harris D (2012). The crisis: The president, the Prophet, and the Shah—1979 and the coming of militant Islam.

[CR20] Hochschild A, Machung A (2012). The second shift: Working families and the revolution at home.

[CR21] Human Rights Watch (2002) “We are not the enemy”, Hate crimes against Arabs, Muslims, and those perceived to be Arab or Muslim after September 11, 2001, vol. 14, No. 6(G). Agency France Press, New York

[CR22] Inman ML, Baronn RS (1996). Influence of prototypes on perceptions of prejudice. J Pers Soc Psychol.

[CR23] Johnston P, Mingst K, Sigelman L (1980). Mirror images in Americans’ perceptions of nations and leaders during the Iranian hostage crisis. J Peace Res.

[CR24] Jones T (2000). Shades of brown: the law of skin color. Duke Law J.

[CR25] Katz I (1991). Gordon Allport’s the nature of prejudice. Political Psychol.

[CR26] King EB, Ahmad AS (2010). An experimental field study of interpersonal discrimination toward Muslim job applicants. Pers Psychol.

[CR27] Klicperová-Baker M, Kost’ál J (2011) Patterns of intolerance. What is behind the attitudes to Roma, gays and immigrant minorities. In: Eurosphere Comparative Studies. Work package 4.3, Report, pp 1–20. http://eurospheres.org/publications/workpackage-reports/

[CR28] Lippi-Green R (1994). Accent, standard language ideology, and discriminatory pretext in the Courts. Lang Soc.

[CR29] McLauchlin T, Pearlman W (2011). Out-group conflict, in-group unity?: exploring the effect of repression on intramovement cooperation. J Confl Resolut.

[CR30] Montagne R (2006) Living in Tehrangeles: L.A.’s Iranian Community. National Public Radio. http://www.npr.org/templates/story/story.-php?storyId=5459468

[CR31] Nail PR, Bedell KE, Little CD (2003). Should president Clinton be prosecuted for perjury? The effects of preference for consistency, self-esteem, and political party affiliation. Personal Individ Differ.

[CR32] Nesbitt RE (2004). The geography of thought.

[CR33] New York Times (2007) Charlie Rose interviews Zbigniew Brzezinski, Henry Kissinger and Brent cowcroft. http://www.nytimes.com/2007/06/18/world/americas/18iht-web-rose.html?pagewanted=all&_r=0

[CR34] NIAC: National Iranian-American Council (2007) NIAC protests dispatch cartoon depicting Iranians as cockroaches. http://www.niacouncil.org/niac-protests-dispatch-cartoon-depicting-iranians-as-cockroaches/

[CR35] Nincic M, Russett B (1979). The effect of similarity and interest on attitudes toward foreign countries. Publ Opin Q.

[CR36] Omeish MS (1999). Muslim students’ perceptions of prejudice and discrimination in American academia: challenges, issues, and obstacles and the implications for educators, administrators, and university officials. Diss Abstr Int Sect.

[CR37] Paige S (2014) Iranian-American perceptions of experienced prejudice and discrimination in the political and social context of the United States of America. A dissertation submitted to the graduate division of the university of Hawai’i at Manoa in partial fulfillment of the requirements for the degree of doctor of philosophy in Psychology. University of Hawaii, Honolulu

[CR38] Peteraf M, Shanley M (1997). Getting to know you: a theory of strategic group identity. Strat Manag J.

[CR39] Porter JR, Washington RE (1993). Minority identity and self-esteem. Ann Rev Soc.

[CR40] Rouhana NN, Fiske ST (1995). Perception of power, threat, and conflict intensity in asymmetric intergroup conflict: Arab and Jewish citizens of Israel. J Confl Resolut.

[CR41] Schneider DJ (2005). The psychology of stereotyping.

[CR42] Siggins P (2002) Racial profiling in an age of terrorism. Santa Clara University

[CR43] Tajfel H, Turner JC, Austing WG, Worchel S (1979). An integrative theory of intergroup conflict. The social psychology of intergroup relations.

[CR44] Waldzus S, Mummendey A, Wenzel M (2004). When “different” means “worse”: in-group prototypicality in changing intergroup contexts. J Exp Soc Psychol.

[CR45] Weaver CN (2008). Social distance as a measure of prejudice among ethnic groups in the United States. J Appl Soc Psychol.

[CR46] Weinfurt KP, Moghaddam FM (2001). Culture and social distance: a case study of methodological cautions. J Soc Psychol.

[CR47] White RK (1970). Nobody wanted war.

[CR48] Wikipedia (2015) List of Iranian-Americans. http://en.wikipedia.org/wiki/List_of_Iranian_Americans (pp. 1-9)

[CR49] Wikipedia (2015) Debra Cagan. http://en.wikipedia.org/wiki/Debra_Cagan

[CR50] Yamagishi T, Mifune N, Liu JH, Pauling J (2008). Exchanges of group-based favors: ingroup bias in the prisoners dilemma game with minimal groups in Japan and New Zealand. Asian J Soc Psychol.

[CR51] Zaman NK (2010). Understanding the experiences of discrimination in Muslim Americans post 9/11: a qualitative study. Diss Abstr Int.

